# Are Intra-operative Forced Air Warming Devices a Possible Source for Contamination During Hand Surgery?

**DOI:** 10.7759/cureus.46287

**Published:** 2023-09-30

**Authors:** Anthony Gemayel, Kyle Flikkema, Germaine Fritz, Daniel Blascak

**Affiliations:** 1 Orthopedic Surgery, New York University (NYU) Langone, New York, USA; 2 Orthopedic Surgery, Beaumont Health, Farmington Hills, USA

**Keywords:** orthopedic surgery, bair hugger, hand surgery, temperature control, surgery, intra-operative hypothermia, forced air warming, contamination

## Abstract

Background

Forced air warming (FAW) devices are routinely utilized in operating rooms for patient temperature control. However, there have been some controversy and conflicting evidence on whether they are a possible source of surgical site infections (SSIs) and contamination.

Methods

A total of 144 petri dishes were randomized to either a control or experimental group (72 in each group). Each trial consisted of six petri dishes in three locations (floor, table, and operative limb). Two dishes at each location were closed sequentially at one hour, two hours, and three hours. Two control and two experimental trials were performed in two separate operating suites with two different FAW devices. The petri dishes were then analyzed for growth for 48 hours. Two culture swabs from each FAW device hose were obtained and analyzed.

Results

None of the culture swabs analyzed showed any growth on blood or chocolate agar culture media. There was no significant difference in bacterial colony-forming units per cubic meter (CFU/m^3^) air between the trial and control groups in each location at one hour of exposure. At two hours of exposure, there was a significantly higher bacterial CFU/m^3^ air in the experimental group in the operative limb. At three hours of exposure, there was a significantly higher bacterial CFU/m^3^ air in the experimental group on the floor. However, overall, there was no difference in bacterial CFU/m^3^ air in both study groups at different times of exposure, incubation, or location.

Conclusion

Our study was unable to identify any statistically significant risk of contamination associated with the use of FAW devices. However, our study design was limited due to the absence of operating room staff during testing. For this reason, we recommend further research into this topic with the use of an active operating room, which includes simulated movement from the surgeon, anesthesia, scrub technician, nursing, and any other operating room staff who may be present during a real operation.

## Introduction

The benefits of a normothermic patient in the operating room are well documented and cannot be overstated. Most operating rooms are kept around 20℃-23℃ [1]. At this temperature, anesthetized patients are unable to regulate their body temperature and are at increased risk of developing hypothermia [2]. Even mild cases of hypothermia in the operating room can lead to adverse events [3]. Maintaining normothermia during surgery has been associated with decreased intra-operative bleeding [4], decreased surgical site infections (SSIs) [5], decreased length of hospital stay [6], and decreased total costs associated with surgery [7].

The methods for maintaining a normothermic patient in the operating room are broadly categorized into three techniques: forced air warming (FAW) devices, conductive warming with blankets, and circulatory warming systems using a closed fluid circuit [3]. More recently, the use of FAW devices has been called into question. The heated airflow from FAW may lead to temperature gradients causing eddies and a disruption of laminar flow within the operating room [8]. As the impedance of the laminar airflow occurs, particulates throughout the operating room air composed of fibers from operative clothing/drapes [9], microbe-laden dust [10], and desquamated skin and respiratory droplets [11,12] are mobilized by air currents and may settle onto the operative field. These contaminants have the potential to cause surgical site infections (SSIs) by, either directly or indirectly, acting as a nidus for microbial attachments and growth [13]. The importance of this concept especially applies to orthopedic surgery, where SSIs occur with relatively low bacterial loads. In fact, multiple studies have demonstrated that as few as 10 bacterial colony-forming units (CFUs) can lead to deep SSIs after joint replacement surgery [14,15].

Despite the potential benefits of maintaining normothermia in the operative suite, there remains significant debate regarding the safety of forced air warming (FAW) devices. The concerns are primarily aimed at two aspects of FAW: the disruption of clean air through thermal eddies and the direct contamination of the sterile field from the air that blows from FAW devices [3]. Legg et al. [16] compared FAW to radiant warming and demonstrated a significant difference in the number of airborne particles and air temperature. In another study, Legg and Hamer [17] showed an increase in particle concentration by 1000-fold for FAW when compared to radiant warming by drawing particles from below the operative table. McGovern et al. [18] also demonstrated that forced air warming causes under-drape air to pass over the surgical drape and onto the surgical site, whereas conductive fabric warming did not, during simulated hip replacement procedures.

To our knowledge, no previous studies have investigated the use of FAW devices during hand surgery. Therefore, the purpose of this study was to determine whether forced air warming devices directly increase microbial contamination around the operative suite during a standard setup for hand procedures. Our null hypothesis stated that FAW would not result in any significant risk of contamination or increase in colony-forming units on the surgical field.

## Materials and methods

Experimental procedure

**Figure 1:**
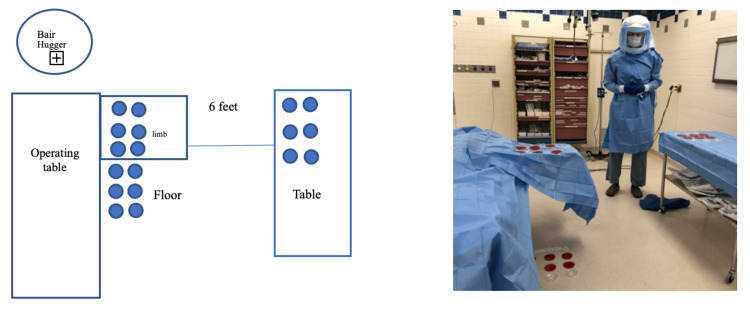
Operative suite setup

Two adjacent operative suites at our institution were utilized to conduct this study. The operative suites had undergone terminal cleaning and had been vacant for approximately 24 hours prior to conducting our study. We performed two control and two trial studies in each operative suite. The Bair Hugger model 775 (3M, St. Paul, MN) was utilized in each room with a new high-efficiency particulate air (HEPA) filter placed. Prior to using each Bair Hugger device, the interior of each nozzle was wiped with two culture swabs (labeled A-D) to be analyzed by the laboratory. Each suite was set up with a standard operative table and hand table. The upper body Bair Hugger drape was placed upon several pillows/blankets and placed on the operative table simulating a patient. The hand table and operative table were then covered with sterile drapes as is routinely done during cases. A table with a sterile drape was then placed adjacent to the hand table, approximately 6 ft away to simulate the sterile back table where instruments are routinely kept. The Bair Hugger machine was then placed at the head of the bed where the anesthesia team, who typically utilizes the Bair Hugger device, would be sitting (Figure 1). We had 144 sterile petri dishes, which were randomly assigned a number and labeled by the investigators. The laboratory technicians were blinded to which study group these dishes belonged to.

Beginning with the control studies, we placed six sterile petri dishes at three different locations: the hand table (limb), the sterile table (table), and the floor directly beneath the hand table (floor). Each control trial was performed for three hours, and two petri dishes were designated for each hour, labeled A and B. An individual performing the study would enter the room and place a sterile hood, gown, and gloves. All petri dishes were opened and closed under sterile conditions to avoid contamination, and the Bair Hugger was not turned on. All six petri dishes at the three different sites were opened simultaneously, and two petri dishes were closed at each location at hours 1, 2, and 3. This procedure was repeated for all control trials in each operative suite. The experimental trials also utilized this same technique with the exception that the Bair Hugger was turned on prior to opening the petri dishes and remained on for the duration of the three-hour study.

All petri dishes and culture swabs were then sent to the laboratory at our institution and incubated at 37℃ for 48 hours. The culture swabs were analyzed using chocolate agar and blood agar plating media. The petri dishes were analyzed for colony-forming units (CFU), and growth was examined at 24 and 48 hours. All CFUs were isolated, and identification was attempted. If isolation was unsuccessful, a Gram stain and catalase reaction were reported.

Statistical analysis

According to the enumerated CFUs at 24 and 48 hours, we determined the number of bacterial air (colony-forming units per cubic meter {CFU/m^3^}) at different exposure (close) times of hour based on the method reported by Fekadu and Getachewu [19]. The equation utilized was as follows: 𝑁=5𝑎×10^4^(𝑏𝑡)^−1^, where N=microbial CFU/m^3^, a=number of colonies per petri dish, b=dish surface area (58 cm^2^), and t=exposure time (minutes).

To evaluate the difference between location (floor, limb, and table) and study group (control and trial) at each exposure (close) time of hour, a regression model, including location, study group, and the interaction term of location and study group, was analyzed at each exposure (close) time. All tests of statistical significance were two-sided with a p value of <0.05 indicating a significant difference. Analysis was performed using PROC MIXED procedure in SAS v9.4 (SAS Institute Inc., Cary, NC).

## Results

There were 48 samples analyzed at each incubation time. Table 1 indicates that there was no significant difference in bacterial CFU/m^3^ air between the trial and control groups in each location at one hour of exposure. At two hours of exposure, there was a significantly higher bacterial CFU/m^3^ air in the trial group in the limb (Table 2). Moreover, at three hours of exposure, there was a significantly higher bacterial CFU/m^3^ air in the trial group on the floor (Table 3). Overall, in the table, there was no difference in bacterial CFU/m^3^ air in both study groups at different times of exposure or at different times of incubation. None of the Bair Hugger device culture swabs analyzed showed any growth on blood or chocolate agar culture media (Table 4).

**Table 1:** Bacterial CFU/m3 air at one hour of exposure for different incubation times For bacterial CFU/m^3^ air, means (standard deviations) were shown CFU/m^3^: colony-forming units per cubic meter

Incubation time	Location	Trial	Control	p value
24 hours	Floor	0 (0)	3.592 (6.651)	0.145
48 hours	Floor	0 (0)	3.592 (6.651)	0.243
24 hours	Limb	1.796 (5.080)	0 (0)	0.461
48 hours	Limb	3.592 (6.651)	3.592 (6.651)	1.000
24 hours	Table	1.796 (5.080)	3.592 (6.651)	0.461
48 hours	Table	3.592 (6.651)	3.592 (6.651)	1.000

**Table 2:** Bacterial CFU/m3 air at two hours of exposure for different incubation times For bacterial CFU/m^3^ air, means (standard deviations) were shown CFU/m^3^: colony-forming units per cubic meter

Incubation time	Location	Trial	Control	p value
24 hours	Floor	2.694 (3.718)	0 (0)	0.129
48 hours	Floor	3.592 (3.840)	0 (0)	0.056
24 hours	Limb	3.592 (7.680)	0 (0)	0.045
48 hours	Limb	3.592 (7.680)	0 (0)	0.056
24 hours	Table	0 (0)	0 (0)	1.000
48 hours	Table	0.898 (2.540)	0 (0)	0.626

**Table 3:** Bacterial CFU/m3 air at three hours of exposure for different incubation times For bacterial CFU/m^3^ air, means (standard deviations) were shown CFU/m^3^: colony-forming units per cubic meter

Incubation time	Location	Trial	Control	p value
24 hours	Floor	3.592 (4.245)	0.599 (1.693)	0.007
48 hours	Floor	4.191 (5.393)	1.197 (2.217)	0.038
24 hours	Limb	0.599 (1.693)	0 (0)	0.573
48 hours	Limb	1.197 (2.217)	1.197 (2.217)	1.000
24 hours	Table	0.599 (1.693)	0 (0)	0.573
48 hours	Table	0 (0)	0.589 (1.693)	0.670

**Table 4:** Bair Hugger device culture swabs BAP, blood agar; CHOC, chocolate agar

Incubation time	Agar type	A	B	C	D
24 hours	BAP	No growth	No growth	No growth	No growth
24 hours	CHOC	No growth	No growth	No growth	No growth
48 hours	BAP	No growth	No growth	No growth	No growth
48 hours	CHOC	No growth	No growth	No growth	No growth

## Discussion

The aim of this study was to identify whether forced air warming devices directly increased microbial contamination around the operative suite during a standard setup for hand procedures. When using an ultra-clean ventilation system, as with the operating suites in our facility, the operating room has varying air velocities resulting in ultra-clean, clean, and semi-clean zones [20]. The periphery of the operating theater is known as the semi-clean zone. Centrally over the surgical table is the ultra-clean zone. Finally, in between these two zones is the clean zone. In our operating suites, the FAW device is located in the ultra-clean zone, situated on the floor near the operating table. The concern is that FAW devices disrupt the surrounding airflow and mobilize particulate matter from the clean zone to the ultra-clean zone.

In our study, we noted an increase in colony count on the floor compared to the control group at hours 2 and 3; however, it was not statistically significant at two hours. Although there was noted to be a statistically significant difference at two hours at the limb, this was not identified at three hours. Additionally, if there was truly a significant difference in the floor at hour 3, we would expect to see an increase in CFU/m^3^. This is concerning for some sort of contamination or other confounding variable. Overall, we were unable to identify any significant difference between the control and trial groups, regardless of location or time.

Despite our inconsistent findings, there is still concern regarding the use of FAW devices during orthopedic surgery. As previously noted, it only requires 10 bacterial colony-forming units (CFUs) to cause a deep surgical site infection after joint replacement surgery [14,15]. In a retrospective study, McGovern et al. [18] showed a significant difference in deep joint infections when using FAW devices. In a more recent pilot study, Kümin et al. [21] showed four deep SSIs when using FAW devices during hemiarthroplasty for the treatment of femoral neck fractures.

There were several limitations in our study. First, we included a very small sample size. Our study only evaluated cultures from 144 petri dishes, which were spread throughout the operative suite. Due to the relatively low number of culture results, we may have missed significant findings that may have otherwise been noticed if more samples were utilized. Second, our study only used two FAW devices in two separate operating rooms. These devices may not directly correlate with all other types of FAW devices in other operative suites. Third, the operating rooms used in our study had undergone a terminal cleaning and had been vacant for approximately 24 hours prior to the study, which may not represent normal operating room conditions. Lastly, and most importantly, our study design did not include the presence of active operating room staff, a patient, or instrument trays. These variables would have ultimately increased foot traffic and particulate matter within the operative suite. For these reasons, our study undoubtedly does not represent the typical working environment of an active, live operating suite, and we are ultimately unsure how these variables may have impacted the results of our study.

## Conclusions

The results of our study showed no statistically significant risk for contamination when using forced air warming devices during a standard setup for hand procedures in the operative suite. However, due to the several limitations noted in our study, we recommend further research using a live, active operating suite, to better direct the use of forced air warming devices during hand surgery.
